# Role of wearable rhythm recordings in clinical decision making—The wEHRAbles project

**DOI:** 10.1002/clc.23404

**Published:** 2020-07-22

**Authors:** Martin Manninger, Jedrzej Kosiuk, David Zweiker, Mario Njeim, Bor Antolic, Bratislav Kircanski, Jacob M. Larsen, Emma Svennberg, Philippe Vanduynhoven, David Duncker

**Affiliations:** ^1^ Division of Cardiology, Department of Medicine Medical University of Graz Austria; ^2^ Helios Clinic Koethen Germany; ^3^ Wilhelminenhospital, 3^rd^ Medical Department for Cardiology and Intensive Care Vienna Austria; ^4^ Division of Cardiology Hotel Dieu de France Hospital, Saint Joseph University Beirut Lebanon; ^5^ University Medical Centre Ljubljana Department of Cardiology Ljubljana Slovenia; ^6^ Clinical Centre of Serbia Pacemaker Centre Belgrade Serbia; ^7^ Department of Cardiology Aalborg University Hospital Aalborg Denmark; ^8^ Department of Cardiology Karolinska Institutet, Karolinska Hospital Stockholm Sweden; ^9^ Department of Cardiology Arrhythmia Clinic Aalst Belgium; ^10^ Hannover Heart Rhythm Center, Department of Cardiology and Angiology Hannover Medical School Hannover Germany

**Keywords:** digital health, digital medicine, wearables

## Abstract

**Background:**

Multiple wearable devices for rhythm analysis have been developed using either photoplethysmography (PPG) or handheld ECG.

**Hypothesis:**

The aim of this survey was to assess impact of these technologies on physicians' clinical decision‐making regarding initiation of diagnostic steps, drug therapy, and invasive strategies.

**Methods:**

The online survey included 10 questions on types of devices, advantages, and disadvantages of wearable devices as well as case scenarios for patients with supraventricular arrhythmias and atrial fibrillation (AF).

**Results:**

A total of 417 physicians (median age 37 [IQR 32‐43] years) from 42 countries world‐wide completed the survey.

When presented a tracing of a regular tachycardia by a symptomatic patient, most participants would trigger further diagnostic steps (90% for single‐lead ECG vs 83% for PPG, *P* < .001), while a single‐lead ECG would be sufficient to perform an invasive EP study in approximately half of participants (51% vs 22% for PPG, *P* < .001).

When presented with a single‐lead ECG tracing suggesting AF, most participants (90%) would trigger further diagnostic steps. A symptomatic AF patient would trigger anticoagulation treatment to a higher extent as an asymptomatic patient (59% vs 21%, *P* < .001). PPG tracings would only rarely lead to therapeutic steps regardless of symptoms.

Most participants would like scientific society recommendations on the use of wearable devices (62%).

**Conclusions:**

Tracings from wearable rhythm devices suggestive of arrhythmias are most likely to trigger further diagnostic steps, and in the case of PPG recordings rarely therapeutic interventions. A majority of participants expect these devices to facilitate diagnostics and arrhythmia screening but fear data overload and expect scientific society recommendations on the use of wearables.

## 1 INTRODUCTION

Wearable devices have rapidly evolved over the last decade allowing a consumer‐driven rhythm analysis on large segments of symptomatic or healthy individuals.

Devices utilize two methods for heart rhythm analysis—photoplethysmography (PPG) or ECG (Table 1).^1^ PPG is based on light emitting and light sensing diodes that estimate heart rate from changes in blood volume caused by peripheral pulsations. Commercially available smartphones provide the technology to record PPG tracings, which is utilized by third‐party applications. Some devices involve automated algorithms that can detect pulse irregularity and notify the consumer regarding a possible arrhythmia. Wearable single‐lead or multiple lead ECG use electrodes that can be hand‐held or implemented in a wristband or a smartwatch. Health and fitness technology is a rapidly evolving market showing a doubling in revenue within the past 5 years.^2^

**TABLE 1:** Used, recommended, and known ECG‐based and PPG‐based device types

	Device name	I use this device	I recommend this device to patients	I recommend this device to colleagues	I have not heard of this device
ECG‐based devices 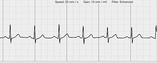	Apple Watch Series 4‐5	16.5%	31.4%	23.7%	6.2%
	Beurer mobile ECG device	5.1%	5.9%	3.8%	75.8%
	imPulse	0.3%	1.8%	0.4%	84.4%
	Kardia Mobile	20.4%	30.9%	26.9%	38.2%
	Kardia Mobile 6L	7.8%	18.4%	15.9%	43.7%
	My Diagnostick	4.2%	3.4%	2.9%	74.0%
	Zenicor‐ECG	4.4%	4.9%	3.8%	72.6%
PPG‐based devices 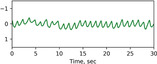	Apple Watch	17.9%	21.4%	17.1%	10.1%
	CardiioRhythm	2.0%	4.3%	2.8%	71.4%
	FibriCheck	4.1%	3.6%	2.8%	75.1%
	Fitbit	9.4%	5.8%	4.7%	44.6%
	HeartRate	1.8%	2.9%	2.1%	76.4%
	Oura Ring	0.3%	0.8%	1.0%	87.2%

The aim of this survey was to assess impact of these technologies on physicians' clinical decision‐making regarding initiation of diagnostic steps, drug therapy, and invasive strategies.

## 2 METHODS

An online questionnaire was prepared using the EHRA Young EP infrastructure and distributed to EHRA Young EP members, members of national electrophysiology (EP) working groups and via social media platforms (Twitter, Facebook). The questionnaire included baseline questions on demographics as well as 10 questions on types of devices, advantages and disadvantages of wearable devices, as well as gaps in evidence. Three case scenarios for (a) a young patient with palpitations, (b) symptomatic atrial fibrillation (AF), and (c) asymptomatic AF were presented to ask for clinical decision‐making. The full questionnaire is available in Supporting Information. Questions were classified as nonmandatory. Responses were excluded if no answer was given at any clinical case scenario or two responses were submitted by one person. In case of missing data, pairwise deletion was performed.

Continuous variables are presented as mean ± SD or median (interquartile range—IQR). Categorical variables are presented as percentages and counts. Questions of clinical decision‐making were compared using Wilcoxon test for dependent and Mann‐Whitney‐*U* test for independent variables. Consensus between respondents was measured using the consensus measure “C,” ranging from 0 (no consensus) to 1 (complete consensus).^3^ A two‐sided *P*‐value of <.05 was considered significant. Statistical analyses were performed using the SPSS 20.0 (IBM, Armonk, New York) and R 3.6.1 (The R Foundation, Vienna, Austria).

## 3 RESULTS

Four hundred and seventeen participants completed the online survey from October 1st to December 31st, 2019. Eleven cases were excluded due to blank input and two cases were excluded due to double entry (matching contact details). The remaining 404 cases were used for the final analysis. Missing data was present in <5% of clinical scenario questions and <12% of other questions.

Median age was 37 years (IQR 32‐43 years). Most participants were EP specialists (32%), followed by cardiologists or cardiology fellows (18.5% each), EP team leaders (15.5%), electrophysiology fellows (12.5%), and doctors or researchers of other professions (3%). Median experience in EP was 5 years (IQR 1‐10 years).

Physicians from 42 different countries participated in the survey, most of them were from Germany, Denmark (15% each), Serbia (14%), France (7%), Spain and Austria (5% each). Six percent of participants were from non‐European countries.

Most of the participants were in a position to take clinical decisions either independently (75%) or under supervision (21%).

### 3.1 Device types

Best known and most recommended ECG devices were the Apple Watch (Apple Inc, Cupertino, California) and Kardia Mobile (AliveCor Inc, Mountain View, California; Table 1).

Most popular PPG devices were the Apple Watch, followed by Fitbit (Fitbit Inc, San Francisco, California), Cardiio Rhythm (Cardiio Inc, Cambridge, Massachusetts), and FibriCheck (Qompium nv, Hasselt, Belgium). The most recommended PPG device was the Apple Watch.

Neither ECG nor PPG devices were reimbursed in the participants' countries (≤2% each).

### 3.2 Interaction between wearable device user and healthcare team

Most participants would prefer remote transmission of the wearable device tracing to a specialized center (34%) over direct presentation to the responsible physician (29%) or physician recommending the device (18%). Only 9 % would leave interpretation of the tracings to third parties.

After data is transmitted to the healthcare team, most participants would prefer that the hospital or clinic nurse (34%), cardiologist (20%), primary care physician (11%), or cardiac electrophysiologist (6%) contacts the patient. Only 19% would prefer that the patient should schedule an appointment.

### 3.3 Patient‐scenarios for clinical decision‐making

**FIGURE 1:**
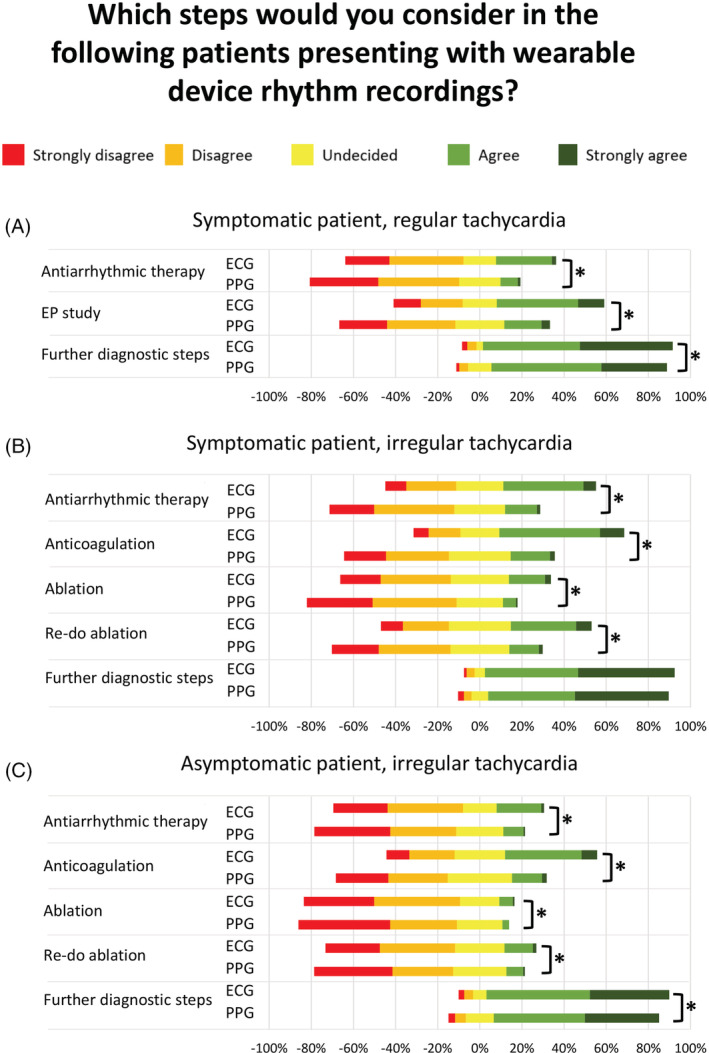
Lickert‐scale on clinical decision‐making in patients with wearable device rhythm recordings. Participants would most likely perform further diagnostic steps in patients with regular, A, or irregular, B, tachycardia recordings and would less likely take other clinical decisions in asymptomatic patients, C, Asterisks indicate significant difference between ECG and PPG recordings (*P* < .001)

In a case example of a young patient with palpitations with on/off‐phenomenon presenting a 30 seconds tracing from a wearable device indicating narrow‐complex tachycardia (in a single‐lead ECG) or regular tachycardia (in a PPG tracing), most participants would trigger further diagnostic steps (90% in single‐lead ECG, C = 0.70, 83% in PPG, C = 0.74, Figure 1A). For half of the participants (51%), a single‐lead ECG would be sufficient to perform an invasive electrophysiological study, while only 22% would indicate an invasive electrophysiological study based on a PPG tracing. Participants would be more reluctant to start antiarrhythmic drug therapy in this patient (28% for single‐lead ECG, C = 0.55, 9% for PPG, C = 0.67).

In a case example of a patient with palpitations presenting a 30 seconds tracing indicating AF, most participants would trigger further diagnostic steps (90% for single‐lead ECG, C = 0.72, 86% for PPG, C = 0.68, Figure 1B). The majority of participants would start anticoagulation (if indicated by CHA_2_DS_2_‐VASc‐Score) based on a single‐lead ECG (59%, C = 0.58), but not on a PPG tracing (21%, C = 0.59).

In a case example of an asymptomatic patient presenting a 30 seconds tracing indicating AF, most participants would also trigger further diagnostic steps (87% for single‐lead ECG, C = 0.71, 79% for PPG, C = 0.68, Figure 1C). Participants would start anticoagulation (if indicated by CHA_2_DS_2_‐VASc‐Score) in 44% (C = 0.57) based on a single‐lead ECG and only 14% (C = 0.59) based on a PPG tracing. In the asymptomatic patient case, participants would be reluctant to start antiarrhythmic drug therapy, perform ablation therapy or re‐do‐ablation based on a single‐lead ECG and PPG tracing.

In general, participants would more likely take clinical actions based on a single‐lead ECG than on a PPG recording (*P* < .001 for every group‐wise comparison except further diagnostic steps in symptomatic patients with AF recording [*P* = 0.08]).

### 3.4 Diagnosis of atrial fibrillation based on a wearable device tracing

**FIGURE 2:**
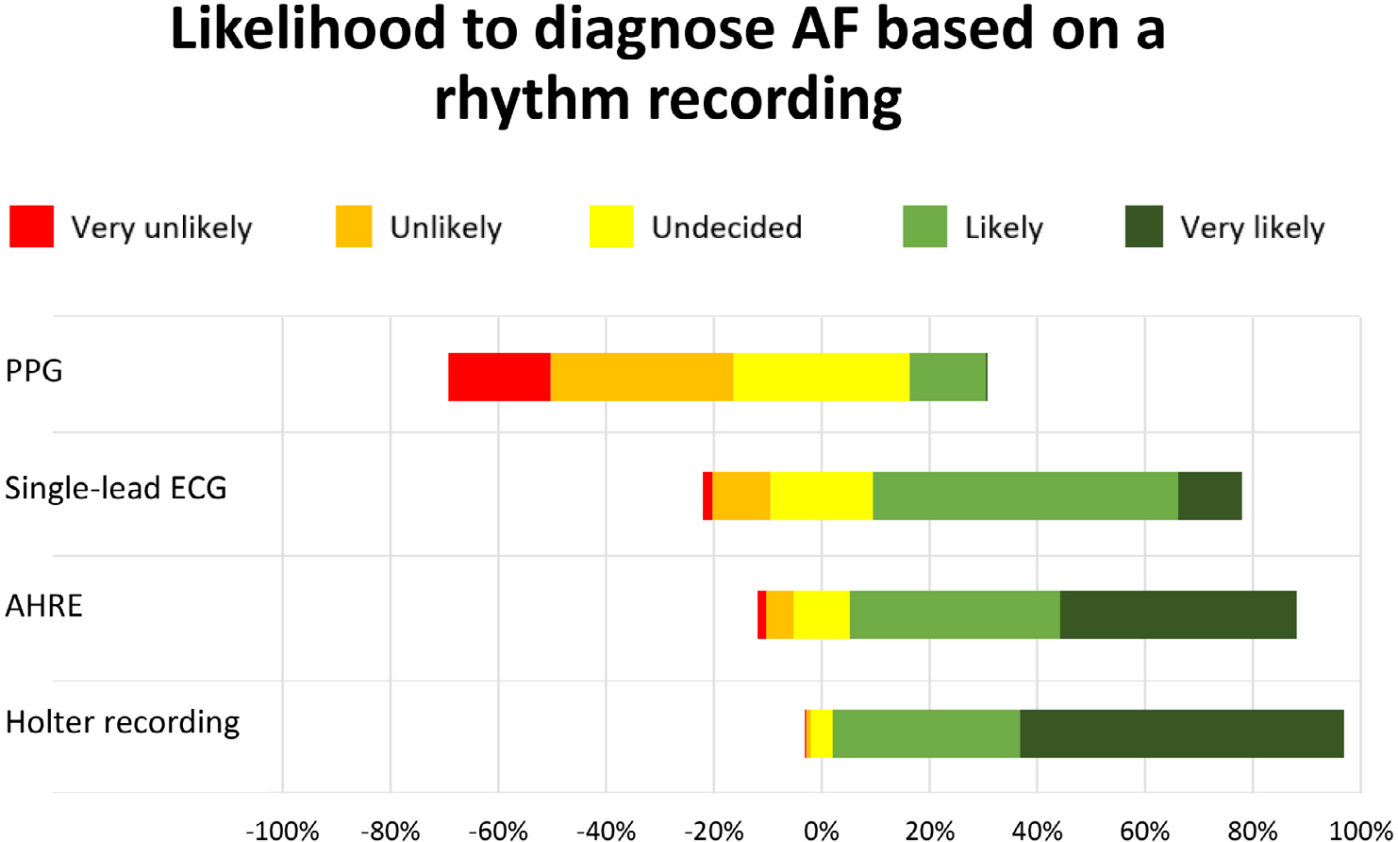
Likelihood of diagnosing atrial fibrillation (AF) based on a 30s recording from Photoplethysmography (PPG), single‐lead ECG, atrial high‐rate episodes (AHRE) from implantable devices and Holter ECG recordings

When presented a 30‐seconds tracing suggesting AF, participants would most likely diagnose AF from a Holter ECG (95% very likely or likely) followed by atrial high rate episodes from an implanted device (83%) or a single‐lead ECG (69%). Only 14% would diagnose AF based on a PPG tracing (Figure 2).

When asked for a cutoff to diagnose AF in a single‐lead ECG tracing, 32% would choose 30 seconds, 10% 1 minute, 2% 5 minutes, 10% 30 minutes, 3% 1 hour, 2% 5.5 hours, and 2% 24 hours. One of four (24.5%) participants would not diagnose AF based on any wearable device tracing. Participants with experience in EP would more likely diagnose AF based on Holter ECGs (mean rank 4.62 vs 4.42, *P* = .006) and single‐lead ECGs (3.83 vs 3.41, *P* < .001) than other cardiologists.

### 3.5 Advantages and disadvantages of wearable devices

**FIGURE 3:**
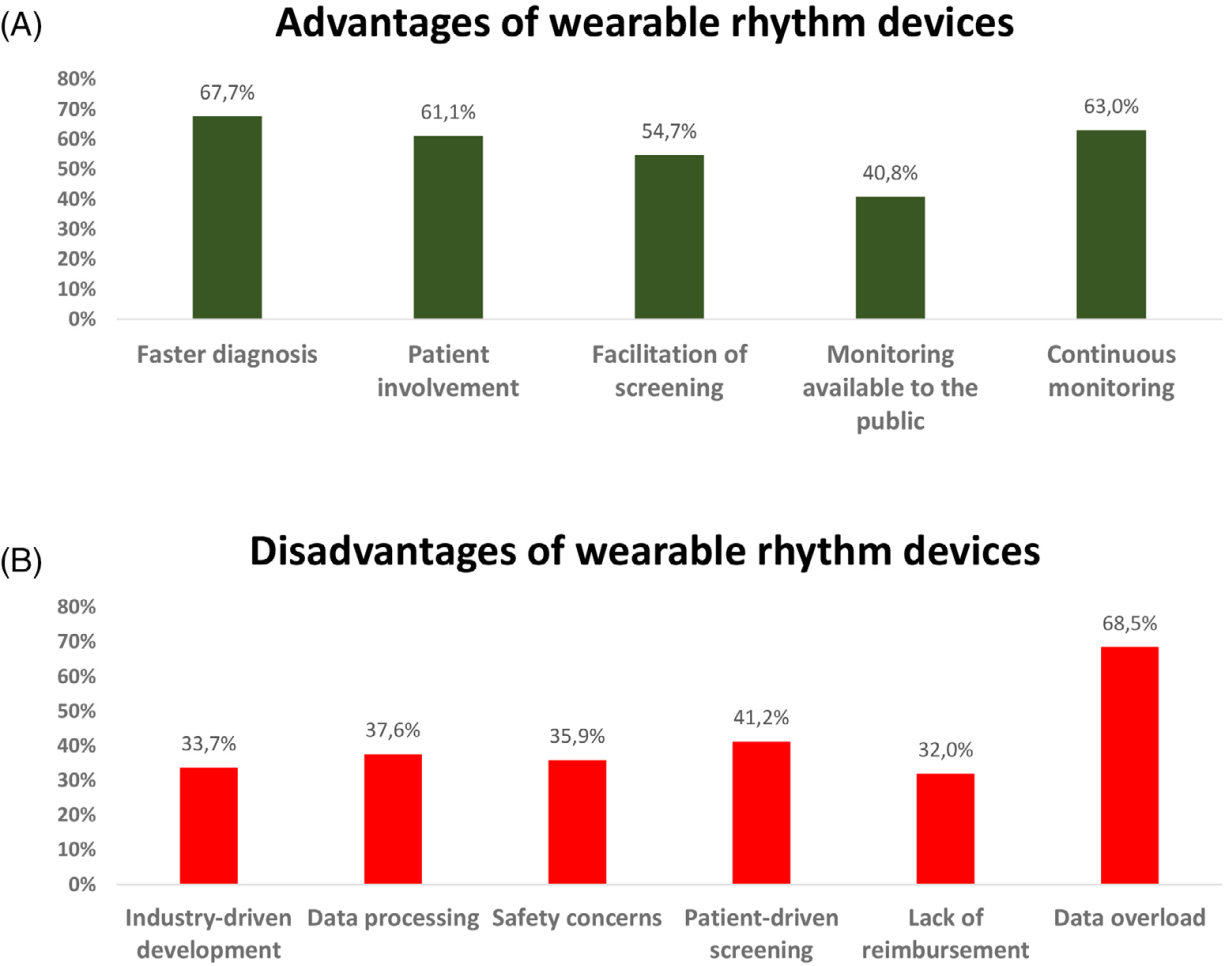
Advantages and disadvantages of wearable rhythm devices (multiple answers possible)

Participants saw the potential of wearable devices mainly in allowing faster diagnosis while the main concern was data overload (Figure 3).

In addition to a consensus document from the scientific society, participants would like to see trials comparing sensitivity and specificity of wearable devices (72%) or a review of validated devices (70%) in order to facilitate clinical decision‐making in daily practice.

Most participants saw the need for specific society recommendations (62%).

## 4 DISCUSSION

Over 400 physicians from more than 40 countries participated in the wEHRAbles project—an initiative of members of EHRA Young EP. This is the first structured survey addressing knowledge and acceptance of wearable rhythm devices as well as relevance of PPG and ECG devices for clinical decision‐making.

This survey demonstrates that physicians:

1. know, use and recommend these novel technologies,
2. would rather perform further diagnostics than take clinical decisions based on these recordings,
3. would rather base clinical decisions on single‐lead ECG than PPG recordings,
4. appreciate the devices' potential in facilitating diagnosis and screening,
5. fear data overload, and
6. call for practical guidance.

### 4.1 Device types

The wearable ECG devices that participants of our survey labeled as most known to them, most available, used and recommended to patients or colleagues are Apple Watch and two AliveCor devices (Kardia Mobile and Kardia Mobile 6 L). These answers reflect the number of published scientific papers and the number of patients included in the studies with these particular devices and current media coverage.^4^, ^5^ Besides Apple Watch and Fitbit, PPG‐based devices were generally less popular among participants of our survey.

### 4.2 Interaction between user and healthcare team

Over the past decade, new wearable devices have allowed consumers instead of providers to take charge of collecting their heart rhythm data, thereby significantly expanding the amount of information collected on large segments of symptomatic or healthy individuals. In addition to data storage, deep‐learning algorithms are now capable of distinguishing between sinus rhythm and arrhythmia (primarily AF) with reasonable accuracy.^6^, ^7^ The user‐driven unselected recording of different parameters challenges the interaction between the potential patient and the healthcare team as there is a fear of data‐overload from otherwise healthy people. However, this fear may be overestimated as screening for AF with a median time of 117 days with PPG‐based tachogram in the Apple Heart Study only resulted in 0.5% notification of irregular rhythm.^4^ The Huawei Study revealed that the rate of false positive alarms decreased with age, suggesting screening for AF could benefit at‐risk populations.^8^

When asked how the data from wearable devices should be shared with the healthcare team, only 9% of the present survey stated that they wanted a third party like the industry to provide the data. However, as a group they were not settled who should receive the data, illustrating the challenges ahead with regards to current positions of the various sectors of the healthcare system in heart rhythm management.

Recently, the Heart Rhythm Society and the Consumer Technology Association has launched a joined guidance paper to the users of wearable technologies, that is, potential arrhythmia patients.^9^ This is an important step to ensure appropriate use of wearable technology, but scientific society guidelines or position papers on wearable heart rhythm monitoring devices are warranted to help the healthcare teams tailor their interaction with the wearable device users but also addressing the established healthcare system's desired requirements to the wearable device industry.

### 4.3 Clinical decision‐making in patients with palpitations

Evaluation of intermittent palpitations and asymptomatic arrhythmias has long presented an unmet need in cardiology.

12‐lead‐ECG remains a cornerstone element for the diagnosis. However, the sporadic and infrequent nature of supraventricular tachycardias (SVTs) makes it difficult to capture an episode on ambulatory monitoring. The 2019 ESC guidelines indicate that mobile recording devices may be required for the diagnosis of SVT.^10^ They specifically highlight that wrist‐worn, optically based heart rate monitors are user‐friendly, but at the same time require an appropriate validation. Most participants of the present survey would refer their patients for an invasive EP study based on a single lead ECG tracing. In a study involving healthy adults, Wang et al reported variable accuracy among different wrist‐worn monitors, all being less accurate than a chest strap‐based electrode containing monitor.^11^

Guidelines for the management of AF state that the diagnosis of AF requires an ECG recording and that episodes of 30s are diagnostic.^12^ Since AF is associated with increased risk of stroke, other morbidity and mortality, opportunistic screening is recommended in patients above 65 years or after transient ischemic attack or ischemic stroke. Methods for AF detection stated in the guidelines are pulse taking, ECG rhythm strip recordings, continuous ECG monitoring and interrogation of implanted cardiac devices. An EHRA consensus document on screening for AF explicitly states new wearable devices as AF screening tools.^13^

Recent studies have shown that AF screening via widely available wearable single‐lead ECGs and PPG tracings is feasible and reasonable.^4^, ^6^, ^13^, ^14^, ^15^, ^16^ The present survey shows that most physicians would perform further conventional rhythm diagnostics when confronted with recordings suggesting AF. Nevertheless, most physicians would consider a wearable device recording sufficient to start anticoagulation. Although sensitivity and specificity of PPG tracings are comparable to single‐lead ECGs, physicians would rather make clinical decisions based on single‐lead ECGs.

When comparing single‐lead ECGs and PPG tracings to conventional Holter recordings and AHRE from implanted cardiac devices, physicians would surprisingly rather diagnose AF based on the latter, although current consensus documents highlight that important questions of diagnostic yield and impact of AHRE episodes remain unanswered.^17^

Although studies on anticoagulation in patients with AF detected by wearable devices are still lacking, physicians in this study agree that anticoagulation should be considered in symptomatic patients when detected by single‐lead ECG. Physicians would be more reluctant to prescribe anticoagulation in asymptomatic patients, although stroke risk in patients with AF is not linked to symptoms of AF.^12^ This finding highlights that further studies and consensus is required on when and whom to anticoagulate based on wearable device rhythm recordings. A new subtype classification of AF may be needed for asymptomatic patients with AF detected by wearable devices in order to allow specific recommendations, comparable to device‐detected AHRE.

### 4.4 Advantages and disadvantages of wearable devices

The possibility of unlimited monitoring resulting in a faster diagnosis is one important advantage of wearable devices. It provides the opportunity to screen large populations. The traditional Holter and event recorders only monitor the heart rhythm for a limited period of time, are uncomfortable to wear and require healthcare staff to properly apply it to the patient. Implantable loop recorders can monitor for years but require an invasive procedure. Therefore, it is not inconceivable that wearables will lead to a reduced use of the current systems and therefore may be cost‐effective.

Data overload is by far the most important disadvantage. Dealing with vast amounts of information coming from technology that might have limited accuracy is a big concern. Especially false positive results will lead to overdiagnosis, over treatment, and more workload for the physician.

The other important disadvantage of wearable devices is that this technology is patient‐driven rather than clinician‐driven. As shown in the Apple Hearth Study and the Huawei study a minority of the participants were over 64 years of age, and in the case of arrhythmia detection would rarely indicate a change of therapy. It is a concern that these technologies mainly reaches the younger, healthier population, leading to an overconsumption of healthcare in otherwise healthy subjects.^4^

### 4.5 Perspectives

It is anticipated that the use of wearable devices will increase from 325 million connected devices in 2016 to 1.1 billion devices by 2021.^18^ As no proper guidance exists for interpretation of wearable device recordings, it is expected that this surge of wearable technology will most likely disrupt the traditional delivery of healthcare.

There is a lack of scientific data making it difficult to choose the right device type for the right patient. Trials comparing the diagnostic properties of the different wearable technologies and the effect on outcome are needed.

There will be a demand for reimbursement, not only of the specific technology, but also of the medical support to process all the generated data.

### 4.6 Limitations

The present survey has limitations attributed to target respondents and questionnaire design. The survey was mainly spread through the scientific network of EHRA Young EP and participation was completely voluntary, therefore being prone to selection bias. Since the survey was distributed by the EHRA Young EP network and had relatively young participants more prone to use wearable technologies, this may reflect a selection bias and needs to be taken into account when interpreting the data. Furthermore, in the presented case scenarios, the preformulated answers may not represent all possible choices that could be made in individual clinical settings.

The current survey focusses on patient‐triggered recordings presented to physicians and does not differentiate between advantages, disadvantages and availability of different types of wearable devices.

## 5 CONCLUSION

Physicians from more than 40 countries are well aware of current wearable rhythm devices and already use and recommend these novel technologies. Tracings from wearable rhythm devices suggestive of arrhythmias are most likely to trigger further diagnostic steps, and in the case of PPG recordings rarely therapeutic interventions. A majority of participants expect these devices to facilitate diagnostics and arrhythmia screening but fear data overload and expect scientific society recommendations on the use of wearables.

## CONFLICT OF INTEREST

Martin Manninger has received speaker honoraria and/or travel grants from Biosense Webster, Abbott, Biotronik, Zoll, Boston Scientific, Daiichi Sankyo, Bayer, Amomed as well as research grants from Biosense Webster. Jedrzej Kosiuk has nothing to declare. David Zweiker has received speaker honoraria and/or travel grants from Daiichi Sankyo and research grants from Boston Scientific. Mario Njeim has received speaker honoraria and/or travel grants from Medtronic, Biosense Webster, Abbott, Biotronik, and Boston Scientific. Bor Antolic has received speaker honoraria and/or travel grants from Biosense Webster, Bayer, Boehringer‐Ingelheim, Medtronic, and Biotronik. Philippe Vanduynhoven has received speaker honoraria and/or travel grants from Abbott, Daiichi‐Sankyo, Bayer, Medtronic, and Boehringer‐Ingelheim. Bratislav Kircanski has received speaker honoraria from Medtronic and travel grants from Medtronic, Biotronik, and Abbott, Boston Scientific. Jacob M. Larsen has nothing to declare. Emma Svennberg has received speaker honoraria from Bayer, Bristol‐Myers Squibb‐Pfizer, Boehringer‐ Ingelheim, Merck‐Sharp and Dohme and Sanofi, as well as institutional research grants from Carl Bennett Ltd and Roche Diagnostics. David Duncker has received speaker honoraria and/or travel grants from Abbott, Astra Zeneca, Biotronik, Boehringer Ingelheim, Boston Scientific, Medtronic, and Zoll.

## Supporting information


**Appendix** **S1**: Supporting Information
