# Supplementary material for: Role of wearable rhythm recordings in clinical decision making—The wEHRAbles project
Source: Clin Cardiol. 2020 Jul 22;43(9):1032–9. doi: 10.1002/clc.23404 (PMC7462183; doi:10.1002/clc.23404)
Supplement: Supplementary file 1 — Appendix S1: Supporting Information [file CLC-43-1032-s001.docx]

**Supplementary material**

**Role of wearable rhythm recordings in clinical decision making – The wEHRAbles project**

Martin Manninger, MD, PhD^1^, Jedrzej Kosiuk, MD^2^, David Zweiker, MD^1,3^, Mario Njeim, MD^4^, Bor Antolic, MD, PhD^5^, Bratislav Kircanski, MD^6^, Jacob Mosgaard Larsen, MD, PhD^7^, Emma Svennberg, MD, PhD^8^, Philippe Vanduynhoven, MD^9^, David Duncker, MD^10^

Affiliations:

1. Division of Cardiology, Department of Medicine, Medical University of Graz; Graz, Austria
2. Helios Clinic Koethen; Koethen, Germany
3. Wilhelminenhospital, 3^rd^ Medical Department for Cardiology and Intensive Care; Vienna, Austria
4. Division of Cardiology, Hotel Dieu de France Hospital, Saint Joseph University, Beirut, Lebanon
5. University Medical Centre Ljubljana, Department of Cardiology; Ljubljana, Slovenia
6. Clinical Centre of Serbia, Pacemaker Centre, Belgrade, Serbia
7. Department of Cardiology, Aalborg University Hospital, Aalborg, Denmark,
8. Karolinska Institutet, Karolinska Hospital, Department of Cardiology; Stockholm, Sweden
9. Arrhythmia Clinic, Department of Cardiology, ASZ Aalst; Aalst, Belgium
10. Rhythmology and Electrophysiology, Department of Cardiology and Angiology, Hannover Medical School, Hannover, Germany

Survey: <https://fr.surveymonkey.com/r/MSXRJPW> (live from October 1st 2019 until December 31^st^ 2019)

| **Question number** | **Question text** | **Answers** |
| --- | --- | --- |
| 1 | Please indicate which of the devices you know or use: ECG-based devices | AliveCor 6L – AliveCor Inc (6-lead ECG) - I have heard of this device |
|  |  | AliveCor 6L – AliveCor Inc (6-lead ECG) - This device is available in my country |
|  |  | AliveCor 6L – AliveCor Inc (6-lead ECG) - I use this device |
|  |  | AliveCor 6L – AliveCor Inc (6-lead ECG) - I would recommend this device to my patients |
|  |  | AliveCor 6L – AliveCor Inc (6-lead ECG) - I would recommend this device to my colleagues |
|  |  | AliveCor 6L – AliveCor Inc (6-lead ECG) - Device-related analyses or interventions are reimbursed in my country |
|  |  | AliveCor 6L – AliveCor Inc (6-lead ECG) - I have not heard of this device |
|  |  | Kardia Mobile – AliveCor Inc - I have heard of this device |
|  |  | Kardia Mobile – AliveCor Inc - This device is available in my country |
|  |  | Kardia Mobile – AliveCor Inc - I use this device |
|  |  | Kardia Mobile – AliveCor Inc - I would recommend this device to my patients |
|  |  | Kardia Mobile – AliveCor Inc - I would recommend this device to my colleagues |
|  |  | Kardia Mobile – AliveCor Inc - Device-related analyses or interventions are reimbursed in my country |
|  |  | Kardia Mobile – AliveCor Inc - I have not heard of this device |
|  |  | Apple Watch Series 4/5 – Apple Inc - I have heard of this device |
|  |  | Apple Watch Series 4/5 – Apple Inc - This device is available in my country |
|  |  | Apple Watch Series 4/5 – Apple Inc - I use this device |
|  |  | Apple Watch Series 4/5 – Apple Inc - I would recommend this device to my patients |
|  |  | Apple Watch Series 4/5 – Apple Inc - I would recommend this device to my colleagues |
|  |  | Apple Watch Series 4/5 – Apple Inc - Device-related analyses or interventions are reimbursed in my country |
|  |  | Apple Watch Series 4/5 – Apple Inc - I have not heard of this device |
|  |  | imPulse - Plessey Semiconductors Ltd - I have heard of this device |
|  |  | imPulse - Plessey Semiconductors Ltd - This device is available in my country |
|  |  | imPulse - Plessey Semiconductors Ltd - I use this device |
|  |  | imPulse - Plessey Semiconductors Ltd - I would recommend this device to my patients |
|  |  | imPulse - Plessey Semiconductors Ltd - I would recommend this device to my colleagues |
|  |  | imPulse - Plessey Semiconductors Ltd - Device-related analyses or interventions are reimbursed in my country |
|  |  | imPulse - Plessey Semiconductors Ltd - I have not heard of this device |
|  |  | MyDiagnostick - MyDiagnostick Medical BV - I have heard of this device |
|  |  | MyDiagnostick - MyDiagnostick Medical BV - This device is available in my country |
|  |  | MyDiagnostick - MyDiagnostick Medical BV - I use this device |
|  |  | MyDiagnostick - MyDiagnostick Medical BV - I would recommend this device to my patients |
|  |  | MyDiagnostick - MyDiagnostick Medical BV - I would recommend this device to my colleagues |
|  |  | MyDiagnostick - MyDiagnostick Medical BV - Device-related analyses or interventions are reimbursed in my country |
|  |  | MyDiagnostick - MyDiagnostick Medical BV - I have not heard of this device |
|  |  | Zenicor-ECG - Zenicor Medical Systems AB - I have heard of this device |
|  |  | Zenicor-ECG - Zenicor Medical Systems AB - This device is available in my country |
|  |  | Zenicor-ECG - Zenicor Medical Systems AB - I use this device |
|  |  | Zenicor-ECG - Zenicor Medical Systems AB - I would recommend this device to my patients |
|  |  | Zenicor-ECG - Zenicor Medical Systems AB - I would recommend this device to my colleagues |
|  |  | Zenicor-ECG - Zenicor Medical Systems AB - Device-related analyses or interventions are reimbursed in my country |
|  |  | Zenicor-ECG - Zenicor Medical Systems AB - I have not heard of this device |
|  |  | Beurer mobile ECG device – Beurer - I have heard of this device |
|  |  | Beurer mobile ECG device – Beurer - This device is available in my country |
|  |  | Beurer mobile ECG device – Beurer - I use this device |
|  |  | Beurer mobile ECG device – Beurer - I would recommend this device to my patients |
|  |  | Beurer mobile ECG device – Beurer - I would recommend this device to my colleagues |
|  |  | Beurer mobile ECG device – Beurer - Device-related analyses or interventions are reimbursed in my country |
|  |  | Beurer mobile ECG device – Beurer - I have not heard of this device |
|  |  | Other (please specify in question 3) - I have heard of this device |
|  |  | Other (please specify in question 3) - This device is available in my country |
|  |  | Other (please specify in question 3) - I use this device |
|  |  | Other (please specify in question 3) - I would recommend this device to my patients |
|  |  | Other (please specify in question 3) - I would recommend this device to my colleagues |
|  |  | Other (please specify in question 3) - Device-related analyses or interventions are reimbursed in my country |
|  |  | Other (please specify in question 3) - I have not heard of this device |
| 2 | Please indicate which of the devices you know or use: Photoplethysmography-based application | CardiioRhythm – Cardiio Inc - I have heard of this device |
|  |  | CardiioRhythm – Cardiio Inc - This device is available in my country |
|  |  | CardiioRhythm – Cardiio Inc - I use this device |
|  |  | CardiioRhythm – Cardiio Inc - I would recommend this device to my patients |
|  |  | CardiioRhythm – Cardiio Inc - I would recommend this device to my colleagues |
|  |  | CardiioRhythm – Cardiio Inc - Device-related analyses or interventions are reimbursed in my country |
|  |  | CardiioRhythm – Cardiio Inc - I have not heard of this device |
|  |  | FibriCheck – Qompium nv - I have heard of this device |
|  |  | FibriCheck – Qompium nv - This device is available in my country |
|  |  | FibriCheck – Qompium nv - I use this device |
|  |  | FibriCheck – Qompium nv - I would recommend this device to my patients |
|  |  | FibriCheck – Qompium nv - I would recommend this device to my colleagues |
|  |  | FibriCheck – Qompium nv - Device-related analyses or interventions are reimbursed in my country |
|  |  | FibriCheck – Qompium nv - I have not heard of this device |
|  |  | HeartRate – Health Preference, LCC - I have heard of this device |
|  |  | HeartRate – Health Preference, LCC - This device is available in my country |
|  |  | HeartRate – Health Preference, LCC - I use this device |
|  |  | HeartRate – Health Preference, LCC - I would recommend this device to my patients |
|  |  | HeartRate – Health Preference, LCC - I would recommend this device to my colleagues |
|  |  | HeartRate – Health Preference, LCC - Device-related analyses or interventions are reimbursed in my country |
|  |  | HeartRate – Health Preference, LCC - I have not heard of this device |
|  |  | Apple Watch – Apple Inc - I have heard of this device |
|  |  | Apple Watch – Apple Inc - This device is available in my country |
|  |  | Apple Watch – Apple Inc - I use this device |
|  |  | Apple Watch – Apple Inc - I would recommend this device to my patients |
|  |  | Apple Watch – Apple Inc - I would recommend this device to my colleagues |
|  |  | Apple Watch – Apple Inc - Device-related analyses or interventions are reimbursed in my country |
|  |  | Apple Watch – Apple Inc - I have not heard of this device |
|  |  | Fitbit – Fitbit Inc - I have heard of this device |
|  |  | Fitbit – Fitbit Inc - This device is available in my country |
|  |  | Fitbit – Fitbit Inc - I use this device |
|  |  | Fitbit – Fitbit Inc - I would recommend this device to my patients |
|  |  | Fitbit – Fitbit Inc - I would recommend this device to my colleagues |
|  |  | Fitbit – Fitbit Inc - Device-related analyses or interventions are reimbursed in my country |
|  |  | Fitbit – Fitbit Inc - I have not heard of this device |
|  |  | Oura Ring – Oura Health Ltd - I have heard of this device |
|  |  | Oura Ring – Oura Health Ltd - This device is available in my country |
|  |  | Oura Ring – Oura Health Ltd - I use this device |
|  |  | Oura Ring – Oura Health Ltd - I would recommend this device to my patients |
|  |  | Oura Ring – Oura Health Ltd - I would recommend this device to my colleagues |
|  |  | Oura Ring – Oura Health Ltd - Device-related analyses or interventions are reimbursed in my country |
|  |  | Oura Ring – Oura Health Ltd - I have not heard of this device |
|  |  | Other (please specify in question 3) - I have heard of this device |
|  |  | Other (please specify in question 3) - This device is available in my country |
|  |  | Other (please specify in question 3) - I use this device |
|  |  | Other (please specify in question 3) - I would recommend this device to my patients |
|  |  | Other (please specify in question 3) - I would recommend this device to my colleagues |
|  |  | Other (please specify in question 3) - Device-related analyses or interventions are reimbursed in my country |
|  |  | Other (please specify in question 3) - I have not heard of this device |
| 3 | Have you used any other devices that were not mentioned in the list above? If yes, please state. | Open-Ended Response |
| You see a young patient with palpitations with on/off-phenomenon in your outpatient clinic. | | |
| 4 | He shows you a 30s recording from a single-lead ECG device indicating regular narrow-complex tachycardia of 170bpm. Would this tracing be sufficient for you to lead to: | Start antiarrhythmic drug therapy |
|  |  | Indication for invasive EP study |
|  |  | Trigger further diagnostic steps |
| 5 | He shows you a 30s recording from a photoplethysmography-device indicating regular tachycardia of 170bpm. Would this tracing be sufficient for you to lead to | Start antiarrhythmic drug therapy |
|  |  | Indication for invasive EP study |
|  |  | Trigger further diagnostic steps |
| You see a patient with palpitations in your outpatient clinic. | | |
| 6 | She shows you a 30s recording from a single-lead ECG device indicating atrial fibrillation. Would this tracing be sufficient for you to lead to: | Start antiarrhythmic drug therapy |
|  |  | Start anticoagulation if indicated by CHA2DS2-VASc-Score |
|  |  | Indication for ablation therapy |
|  |  | Indication for re-do ablation in previously diagnosed and ablated AF |
|  |  | Trigger further diagnostic steps (e.g. 24h Holter ECG) |
| 7 | She shows you a 30s recording from a photoplethysmography device showing absolute arrhythmia. Would this tracing be sufficient for you to lead to: | Start antiarrhythmic drug therapy |
|  |  | Start anticoagulation if indicated by CHA2DS2-VASc-Score |
|  |  | Indication for ablation therapy |
|  |  | Indication for re-do ablation in previously diagnosed and ablated AF |
|  |  | Trigger further diagnostic steps (e.g. 24h Holter ECG) |
| You see an asymptomatic patient in your outpatient clinic who had a warning from a wearable device suggesting consultation of a specialist. | | |
| 8 | She shows you a recording of at least 30s from a single-lead ECG device indicating atrial fibrillation. Would this tracing be sufficient for you to lead to: | Start antiarrhythmic drug therapy |
|  |  | Start anticoagulation if indicated by CHA2DS2-VASc-Score |
|  |  | Indication for ablation therapy |
|  |  | Indication for re-do ablation in previously diagnosed and ablated AF |
|  |  | Trigger further diagnostic steps (e.g. 24h Holter ECG) |
| 9 | She shows you a 30s recording from a photoplethysmography device showing absolute arrhythmia. Would this tracing be sufficient for you to lead to: | Start antiarrhythmic drug therapy |
|  |  | Start anticoagulation if indicated by CHA2DS2-VASc -Score |
|  |  | Indication for ablation therapy |
|  |  | Indication for re-do ablation in previously diagnosed and ablated AF |
|  |  | Trigger further diagnostic steps (e.g. 24h Holter ECG) |
| 10 | How likely would you establish the diagnosis of atrial fibrillation based on a 30s tracing recorded from the following devices? | photoplethysmography event recorder tracing |
|  |  | single-lead event recorder ECG |
|  |  | 3-channel Holter recording |
|  |  | AHRE episodes on implantable dual chamber device |
| 11 | How long would the tracing of a photoplethysmography or single-lead ECG device have to be so that you would establish the diagnosis of atrial fibrillation? | 30s |
|  |  | 1min |
|  |  | 5min |
|  |  | 30min |
|  |  | 1h |
|  |  | 5.5h |
|  |  | 24h |
|  |  | I would not diagnose AF based in these tracings |
|  |  | Other (please specify) |
| 12 | In addition to a consensus document from your scientific society, which further documents or projects would you like to see to facilitate your clinical decisions regarding recordings from wearable devices? (check all that apply) | Review of all validated devices and their potential role in clinical practice |
|  |  | Trial comparing sensitivity and specificity of every device in comparison to gold standard |
|  |  | Collection of exemplary and educative tracings from PPG and single lead ECG devices |
|  |  | Clinical decision-making tools (e.g. flow charts for further steps required after receiving PPG tracing) |
|  |  | EHRA seal of approval for wearable devices |
|  |  | More education (e.g. webinars) for clinical decision-making |
|  |  | Other (please specify) |
| 13 | In your opinion, what are the biggest advantages of these novel technologies for event recording? (check all that apply) | faster diagnosis |
|  |  | patient involvement |
|  |  | facilitation of screening |
|  |  | making monitoring devices available to the public |
|  |  | continuous monitoring opportunities |
|  |  | Other (please specify) |
| 14 | In your opinion, what are the biggest disadvantages of these novel technologies for event recording? (check all that apply) | Industry-driven development |
|  |  | Industry-driven data processing |
|  |  | Data safety and privacy concerns |
|  |  | Patient-driven instead of clinician-driven screening |
|  |  | Lack of reimbursement |
|  |  | Data overload |
|  |  | Other (please specify) |
| 15 | What additional steps are lacking to encourage you to prescribe wearable devices to your patients? (check all that apply) | I have no reservation recommending those devices without the need for any additional step |
|  |  | Scientific society recommendations regarding the use of wearable devices |
|  |  | Legislations clarifying physician's liability when they recommend wearable devices |
|  |  | Paramedical support to help receive and manage data from wearable devices |
|  |  | Patient reimbursement for acquisition of a wearable device |
|  |  | Physician fee compensation for reviewing and managing data provided by wearable devices |
|  |  | Other (please specify) |
| 16 | In your opinion, how should the data provided by wearable devices best be shared with the health care team? | remote transmission to a hospital or clinic nurse who will triage it and send it to the responsible physician |
|  |  | data should be kept with the patient and presented to the responsible physician at the next patient-physician clinic encounter |
|  |  | a third party (independent from the physician's health care institution) should receive the data, triage it and transmit to the physician |
|  |  | the primary physician who recommended the wearable device should be the first party to receive the data electronically |
|  |  | Other (please specify) |
| 17 | After the data is transmitted to the health care team who should be the first respondent contacting the patient with a management plan? | Hospital or clinic nurse after consulting with responsible physician |
|  |  | Primary care physician |
|  |  | Cardiologist |
|  |  | Cardiac electrophysiologist |
|  |  | The patient should schedule a clinic appointment with the responsible physician |
|  |  | Other (please specify) |
| 18 | Please indicate your age | Open-Ended Response |
| 19 | In which country is your centre based? | Response |
|  |  | Other (please specify) |
| 20 | Primary working environment | Response |
|  |  | University hospital |
|  |  | Specialized public cardiology center |
|  |  | District/community hospital |
|  |  | Private hospital |
|  |  | Private practice |
|  |  | Other (please specify) |
| 21 | Actual working position | Cardiologist – EP / CP team leader |
|  |  | Cardiologist – EP / CP specialist |
|  |  | Cardiologist |
|  |  | EP fellow |
|  |  | Cardiology fellow |
|  |  | Other (please specify) |
| 22 | Working experience: For how many years are you involved in EP? | Open-Ended Response |
| 23 | Are you in a position to decide on diagnostic and therapeutic steps in arrhythmia patients ? | Yes |
|  |  | Yes, under supervision |
|  |  | No |
